# Superior vena cava syndrome due to hyper-homo-cysteinemia: A rare case report

**DOI:** 10.6026/9732063002001305

**Published:** 2024-10-31

**Authors:** Reemu Bansal, Shreya Krishna, Vanshikaa Thakran, Harsh Chandulal Shingala, Rishabh Jain, Sarita Sunil Chammanam, Ravi Sangoi, Krisha Jain

**Affiliations:** 1 Department of Neurosurgery, Marengo Asia Hospital, Faridabad, Haryana, India; 2 Department of Acute Medicine, Sri Ramachandra Institute of Higher Education and Research, Chennai, India; 3 Medical officer, Promestha Clinic, 1st Floor ILD Trade Centre, Sector 47, Gurugram, Haryana; 4 SHO, HCG Hospital, Rajkot, Gujarat, India; 5 Cumberland Infirmary, North Cumbria Integrated Care NHS Foundation Trust, United Kingdom; 6 Department of General Medicine, JSS Academy of Higher Education and Research, Mysore, India; 7 Department of Internal Medicine, Government Medical College and General Hospital, Baramati, Pune, Maharashtra, India

**Keywords:** Superior vena cava syndrome, extra-luminal compression, malignancy, intraluminal obstruction, thrombosis

## Abstract

Superior vena cava (SVC) syndrome (SYN) is a relatively frequent complication observed in patients with malignancies and is frequently characterized as a medical emergency. In most instances, it arises from mechanical obstruction of the SVC caused by extraluminal compression from primary intrathoracic malignancies. Intraluminal obstruction (OB) resulting from thrombosis (TMBO) may also lead to the manifestation of symptoms and signs associated with the syndrome. Clot-related SVC OB is primarily linked to the presence of indwelling central venous catheters and pacemaker leads. However, data shows that this is due to hyper-homo-cysteinemia (HY-HM-CT).

## Background:

Superior vena cava (SVC) syndrome (SYN) is commonly associated with the obstruction of the larger mediastinal veins (MS-V) due to factors such as malignant or benign tumors, MS fibrosis, or trauma. [1] Studies have shown that, thrombosis (TMBO) of the VC frequently occurs as a complication associated with the utilization of indwelling intravenous catheters (IV-CAT) in the contexts of hemodialysis (HM-DA), parenteral nutrition(P-NT) or chemotherapy [1, 2]. The majority of the time, this happens because of the compression or invasion of the SVC by MA masses (such as a tumor or an enlarged lymph node), while TMBO is the cause of this condition less often. In addition to this, a study has shown that, bronchogenic carcinoma (BCH-CAR) is the most prevalent cause of a syndrome that develops gradually between the spleen & the VC. Other malignancies, including lymphoma (LMPH), thymoma & germ cell tumors (GCT) are responsible for the illness [3]. Conversely, in cases of acutely developed SVC-SYN, a benign etiology should be considered as the primary suspicion. In the contemporary landscape of medical science, there has been a notable escalation in the utilization of interventional procedures, including central venous catheter insertion, implantable cardiac defibrillator placement and cardiac pacemaker implantation. This trend has contributed to a significant increase in the incidence of superior VC-SYN of benign etiology, primarily due to its predisposition to TMBO [3, 4].

Historically, infectious lesions were prevalent causes, however, in contemporary practice, malignancy & the utilization of IV devices & cardiac pacemakers have emerged as the primary contributors [5, 6]. Studies have shown that, lung cancer (LC) & LMPH account for over 80% of SVC-SYN cases attributed to malignancy (MLG) [5, 6]. Unlike external compression resulting from MLG, the occurrence of intraluminal metastatic obstruction (ITL-MS-OB) leading to TMBO of the SCV is uncommon. The association between BC & TMBO has been established over time, suggesting that cancer may function as a prothrombotic condition (PTMB-CN) [7]. Cancer is linked to a heightened risk of venous thromboembolism (V-TE), influenced by various tumor-related factors such as histological type, primary tumor site, disease stage, and the timing of the event post-diagnosis. Additionally, patient-related factors including age, comorbid conditions like obesity or pulmonary disease, thrombophilia, and immobilization play a role. Treatment-related factors also contribute, including the use of central venous catheters, recent major surgeries, chemotherapy, and transfusions [8].

Moreover, the presence of mild hyperhomocysteinemia (HY-HM-CT) has been identified as a prominent risk factor for atherosclerosis and vascular disease. However, until recently, it was unclear if mild HY-HM-CT is also a risk factor for venous thrombosis. In classic homocystinuria, 50% of the vascular problems are of venous origin [9]. It is basically a disorder of methionine metabolism which may be result of hereditary defect in the enzyme which leads to vitamin deficiency [10].

## Case presentation:

A postmenopausal Asian lady who was 49 years old and had a history of migraines reported that she had difficulty breathing when she exerted herself and that she had sudden edema in her neck, face and both upper limbs(U-L) (left and right). The swelling began gradually and finally expanded to the upper arm(U-A), hands & forearm of the right side of the body. From there, it eventually went to the U-L, neck, and face of the left side. In addition to being solid, the edema did not pit. The ulnar portion of the forearm & 4th to 5th finger were notably affected by the numbness and severe pin-prick sensations that she experienced in both of her U-A. Furthermore, she had difficulties swallowing solid meals and suffered from throat soreness for a period of 15 days. In the most recent instance, she has been suffering with a dry cough for the last 2 days. This cough was more severe while she was lying down, but it was soothed when she was seated.

Upon admission, the patient presented with an axillary temperature of 97.6°F, a heart rate of 108 beats per minute, oxygen saturation of 98% on room air, and a blood pressure (BP) reading of 110/80 mmHg. The general physical examination reveals pallor, while the local examination notes asymmetrical edema on the face, neck & both U-L, with the right side more affected than the left. During the systemic examination, the chest assessment revealed that the patient exhibited dyspnea and was utilizing accessory muscles for respiration. Upon release of pressure, the anterior chest wall superficial veins expand & create a downward filling pattern. The cardiovascular examination reveals an absence of wheezes or crackles in the chest. The jugular venous pressure (JVP) is non-appreciable, with normal S1 & S2 heart sounds. There are no additional noises or murmurs detected. The neurological assessment indicates that the patient is alert, attentive, and exhibits no focal deficits. The abdominal examination reveals a soft consistency with no tenderness & not organomegaly. After referring her to the gynecology outpatient department, we conducted a breast examination, which revealed no skin abnormalities or prominent veins suggestive of breast cancer (BC).

The laboratory results showed that the patient is anemic with levels of 34 mg/dL of blood urea nitrogen, 0.9 mg/dL of creatinine, 9 g/dL of hemoglobin, and 96 U/L of aspartate aminotransferase. A 2D echocardiogram revealed a left ventricular ejection fraction (LVEF) of 60%, a normal chamber, and a normal right ventricular systolic function (RVSF). In light of the swelling over the upper part of the body, an X-ray of the chest reveals haziness in both CP angles and significant bronchovascular vascular marks on both sides. CECT chest size revealed acute pulmonary (A-PM) V-TE with significant thrombus in the SVC up to cavo-arterial junction and right & left brachiocephalic veins as shown in Figure 1 - see PDF.

Here, the red arrow shows soft tissue density in the svc with contrast flowing around the periphery consistent with acute svc thrombosis. The thrombus has extended to the azygous vein. Linear filling defects are observed in the right subclavian (R-SBC) & internal (IT) JV, characterized by a slightly narrowed caliber indicative of chronic TMBO. There are several swollen, evenly enhancing round to oval nodes on both sides of the body in the prevascular, upper and lower paratracheal & subcarinal areas. The biggest node is 28 mm by 20 mm and is in the right axillary area. Numerous tortuous & prominent collateral channels are observed in the left side of the neck, the left perihilar region, and along the anterior chest wall. A comprehensive profile was conducted to assess the underlying cause of thrombus thrombophilia. The results indicated protein C levels at 68, free protein S at 84, antithrombin at 112 and elevated homocysteine levels at 24.10. Subsequently, genetic testing was performed to assess the methylenetetrahydrofolate reductase (MTHFR) gene mutation. Qualitative PCR analysis revealed the presence of a heterozygous mutation. Furthermore, the APLA panel indicates a negative result for lupus, while the ANA test shows a positive result. The cardiolipin level is recorded at less than 9.37, glycoprotein IGM at 5.78 and glycoprotein IgG at less than 1.80. A speckled pattern with a ratio of 1:80 was completed. Subsequently, ECG and troponin levels were assessed, revealing no indications of myocardial ischemia.

During her hospital stay, her symptoms progressed, resulting in significant dyspnea, necessitating her admission to the ICU. Treatment started with intravenous heparin, administering a bolus of 10,000 units (equal to 80 units/kg).thereafter at a rate of 1200 units per hour (18 units per kilogram per hour). APPT monitoring was established & conducted every 4 hours as to sustain APTT 2.5-3 times the U-L of the standard range. Subsequently, the therapy was altered to include LMWH (80 mg of INJ CLEXANE subcutaneously twice day) in conjunction with folic acid and vitamin B12 intake to lower homocysteine levels. Her symptoms significantly improved with the medication, and she has been released home on enoxaparin and a mix of folate, B12, and B6 vitamins. She has not encountered any more thrombosis over the four-month follow-up period and is in good health.

## Discussion:

According to the American College of Physicians, there are over 500,000 occurrences of V-TE that occur annually in the United States alone, and up to 200,000 of those cases result in mortality [11]. The Turkish research that was conducted not too long ago offered proof that SVC and BCV thrombosis develops as a consequence of specific identified risk factors, the most prominent of which are malignancy, thrombophilia, chronic diseases, or iatrogenic situations following peripheral venous line [12]. Surgical reconstruction techniques, which involve the use of vein grafts or the interposition of an e-PTFE tube, are the most commonly used methods for treating stenosis of the spleen [13, 14]. If surgical reconstruction is performed, the patency of bypass grafts may continue for ten years or possibly beyond this timeframe [14, 15]. Within the realm of new therapeutic techniques, angioplasty and the use of intraluminal stents have shown their effectiveness [15, 16].

A study has shown that, severe HY-HM-CT, referred to as homocysteinuria, is a significant risk factor for the onset of atherosclerotic disease (AS-D) and TE [17]. A not more recent study has demonstrated a correlation between mildly elevated homocysteine levels and an increased risk of arterial occlusive disease (AT-OC-D) [18]. Additionally, evidence indicates that mild HY-HM-CT serves as a significant risk factor for the recurrence of V-TMBO [19, 20]. Another study showed that, SVC syndrome caused by intravascular thrombosis suggesting that RA may be the origin of the hypercoagulable condition in that instance [21]. Van den Brink *et al.* described in their study that, SVC syndrome in a patient with SLE and long-standing typical rheumatoid arthritis. The cause was external compression of the SVC by mediastinal lymphadenopathy [22].

In addition to above, according to a study HY-HM-CT was found significant risk factor for patients younger than 40 years of age [23]. While another study proved that 20 to 70 years of patients are at risk [24]. The results of the past studies support our hypothesis.

## Conclusion:

It is advisable to conduct screening for mild HY-HM-CT using a straightforward methionine loading test in young patients who exhibit thromboembolic events or have atherosclerotic disease. Additionally, bypass reconstruction in carefully chosen patients with SVC-SYN continues to be a viable therapeutic approach when stenting is unsuccessful.
